# A Novel Efficient Piscine Oral Nano-Vaccine Delivery System: Modified Halloysite Nanotubes (HNTs) Preventing Streptococcosis Disease in Tilapia (*Oreochromis* sp.)

**DOI:** 10.3390/vaccines10081180

**Published:** 2022-07-25

**Authors:** Ansaya Pumchan, Udom Sae-Ueng, Chaiya Prasittichai, Soranuth Sirisuay, Nontawith Areechon, Sasimanas Unajak

**Affiliations:** 1 Department of Biochemistry, Faculty of Science, Kasetsart University, 50 Ngam Wong Wan, Chatuchak, Bangkok 10900, Thailand; ansaya.pu@ku.th; 2 Kasetsart Vaccines and Bio-Product Innovation Centre, Kasetsart University, 50 Ngam Wong Wan, Chatuchak, Bangkok 10900, Thailand; 3 National Center for Genetic Engineering and Biotechnology (BIOTEC), National Science and Technology Development Agency (NSTDA), 113 Thailand Science Park, Phahonyothin Road, Khlong Nueng, Khlong Luang, Pathum Thani 12120, Thailand; udom.sae@biotec.or.th; 4 Department of Chemistry, Faculty of Science, Kasetsart University, 50 Ngam Wong Wan, Chatuchak, Bangkok 10900, Thailand; fscicyp@ku.ac.th; 5 Department of Aquaculture, Faculty of Fisheries, Kasetsart University, 50 Ngam Wong Wan Road, Chatuchak, Bangkok 10900, Thailand; ffissns@ku.ac.th (S.S.); ffisnwa@ku.ac.th (N.A.)

**Keywords:** halloysite nanotube (HNT), oral delivery system, nano-vaccine delivery system, streptococcosis, tilapia

## Abstract

Generally, the injection method is recommended as the best efficient method for vaccine applications in fish. However, labor-intensive and difficult injection for certain fish sizes is always considered as a limitation to aquatic animals. To demonstrate the effectiveness of a novel oral delivery system for the piscine vaccine with nano-delivery made from nano clay, halloysite nanotubes (HNTs) and their modified forms were loaded with killed vaccines, and we determined the ability of the system in releasing vaccines in a mimic digestive system. The efficaciousness of the oral piscine vaccine nano-delivery system was evaluated for its level of antibody production and for the level of disease prevention in tilapia. Herein, unmodified HNTs (H) and modified HNTs [HNT-Chitosan (HC), HNT-APTES (HA) and HNT-APTES-Chitosan (HAC)] successfully harbored streptococcal bivalent vaccine with inactivated *S. agalactiae*, designated as HF, HAF, HCF and HACF. The releasing of the loading antigens in the mimic digestive tract demonstrated a diverse pattern of protein releasing depending on the types of HNTs. Remarkably, HCF could properly release loading antigens with relevance to the increasing pH buffer. The oral vaccines revealed the greatest elevation of specific antibodies to *S. agalactiae* serotype Ia in HCF orally administered fish and to some extent in serotype III. The efficacy of streptococcal disease protection was determined by continually feeding with HF-, HAF-, HCF- and HACF-coated feed pellets for 7 days in the 1st and 3rd week. HCF showed significant RPS (75.00 ± 10.83%) among the other tested groups. Interestingly, the HCF-treated group exhibited noticeable efficacy similar to the bivalent-vaccine-injected group (RPS 81.25 ± 0.00%). This novel nano-delivery system for the fish vaccine was successfully developed and exhibited appropriated immune stimulation and promised disease prevention through oral administration. This delivery system can greatly support animals’ immune stimulation, which conquers the limitation in vaccine applications in aquaculture systems. Moreover, this delivery system can be applied to carrying diverse types of biologics, including DNA, RNA and subunit protein vaccines.

## 1. Introduction

To overcome pathogenic outbreaks in fish farming, the most efficient method to control infectious diseases instead of antibiotics and chemical therapeutics is vaccination. Up to now, fish vaccines can be developed using various approaches, including conventional and alternative technologies. In the case of conventional fish vaccines, they have been generally developed depending on the live attenuated vaccine and the inactivated whole-cell vaccine that notably provide high efficacy for disease protection [1]. Remarkably, most of the currently commercialized and licensed vaccines belong to this group. Moreover, alternative vaccines are composed of subunit vaccines and nucleic acid vaccines (DNA- and RNA-based vaccines) that may be expensive to originate but can potentially overcome the limitations of conventional vaccines [2,3]. Although there are various routes for administering the formulated vaccines into fish including immersion, oral and injection methods, however, most of the developed vaccines are administered through the injection method. Despite this route generally providing significant potency, it also generates some drawbacks and limitations, for example, stimulating fish stress, creating difficulty in handling small fish and requiring labor for large-scale farmed fish [4]. To overcome these restricted conditions, immersion and oral administrations have been considered for delivering streptococcal vaccines. Both are more generally applicable than injection but are more complex and difficult for successful development. In the case of the immersion vaccine, it is commonly conducted in two ways, as the dip and bath vaccinations are appreciated for the large-scale vaccination of small fish [5]. However, the efficacy of most immersion vaccines is still low to moderate, owing to various points such as the dose of the antigen, the duration of immersion, temperature, mucosal integrity, fish size, osmolarity, etc. [6]. The optimization of all these variations may be quite laborious, and the mass production of immersion vaccines also has high costs. Additionally, the mechanism of antigen uptake after immersion immunization is still unclear [7]. Therefore, an ideal alternative vaccination to oral vaccination should be investigated more.

To date, the development of oral vaccines is a crucial strategy for their promising vaccination ability in massive, farmed fish. The first oral vaccine dates back to 1942 for trout protection against *Bacterium salmonicida* [8]. Since then, the investigation of oral vaccine regimes against fish pathogens has been heavily studied. However, most current oral formulations can provide only short-term and low protection [9]. Today, a lot of commercially oral vaccines are already available for salmonid species. On the other hand, only several countries, which are mostly Norway, Scotland and Chile, use them practicably [10]. Most likely, due to their weak immunizations, they usually need to be combined with other boosters or vaccinations for enhancing strong and long-term prevention throughout the fish production cycle [11]. Consequently, this combined regime may increase the budgets of farmers and induce more stress for fish. As a result, more rational designs of highly potent oral vaccines have been steadily studied. One approach to ensure the practicality of oral vaccines in the actual field is by incorporating vaccines with suitable delivery systems.

In the past decade, investigations for dietary vaccines using “nano” carriers have garnered considerable attentions. Both synthetic materials (poly-L-lactic acid and polyethylene glycol) and natural materials (alginate, chitosan and cellulose derivatives) have been utilized for biologic carriers [12,13,14]. However, to imitate their unpredictable toxicity to the target host, one of the natural nanoparticles of halloysite nanotubes (HNTs) was considered in this recent study. HNTs, Al_2_Si_2_O_5_(OH)_4_·nH_2_O, are hollow tubular nano clay with an internal diameter of 15–50 nm, an external diameter of 50–80 nm and a length of approximately 100–1000 nm [15]. HNTs have a peculiar feature, i.e., a dissimilar charge between the outer and inner surfaces, owing to their different silica and alumina components [16]. They are also environmentally friendly, biocompatible and have a low cost. In addition, due to their hollow shape, they can promote encapsulated efficacy and can also help to protect susceptible biological molecules through the host’s delivering tract [17]. So far, a variety of advantageous applications based on native HNTs has been researched, including biologic nanocarriers [18,19,20,21,22]. Nevertheless, several studies have suggested that functionalized HNTs manufactured by grafting their surfaces with some substances can enhance their desirable characteristics for use in drug delivery systems [23,24].

*S. agalactiae*, causing the infectious streptococcosis disease in mostly marine and freshwater fish, is one of the harmful pathogens spreading throughout the world, particularly in Southeast Asia, including Thailand [25]. Various forms of *S. agalactiae* vaccines utilized in tilapia have been drastically developed, including attenuated vaccines [26,27], inactivated vaccines [28,29], subunit vaccines [30,31] and DNA vaccines [32,33]. However, most of them were generated for injection administration and may not be practical for large-scale fish farming. Moreover, there is limited literature on the HNT loading vaccine’s platform to facilitate sustainable aquaculture. In this study, the model vaccine of *S. agalactiae* (bivalent streptococcosis vaccine) was loaded on bare HNTs and surface-modified HNTs. We chose amino-silane, chitosan and a combination of amino-silane and chitosan as the surface decorating the HNTs due to their compatibility with fish tissues [34]. The vaccine release profiles on both un-modified and modified HNTs were studied in an environment mimicking the digestive system in fish. The vaccine-trapped HNTs and surface-modified HNTs were subsequently incorporated with feed pellets to form oral vaccinations in tilapia. Finally, the efficacy of each oral *S. agalactiae* vaccine was evaluated and compared with the traditional injection method.

## 2. Materials and Methods

### 2.1. Materials

Unmodified halloysite (H) was purchased from Sigma-Aldrich, and other modified halloysites, including HNTs-APTES (HA), HNTs-Chitosan (HC) and HNTs-APTES-Chitosan (HAC) were fabricated as previously reported [34]. *S. agalactiae*-free tilapia (*Oreochromis* sp.) were obtained from a Charoen Pokphand Foods (CPF) Farm in Thailand. All in vivo experiments were conducted under guidelines approved by the National Research Council of Thailand. Experimental tilapias were anesthetized with clove oil to reduce stress during vaccination and challenge analysis.

### 2.2. Preparation of the StrepKU-1 Vaccine

In this study, the StrepKU-1 (bivalent streptococcosis vaccine) vaccine was used as the vaccine model for the HNT delivery system. Briefly, *S. agalactiae* (serotype Ia and III) was cultured in BHI broth at 30 °C for 16–20 h. After centrifugation at 3300× *g* for 10 min, the harvested bacterial cells were washed thrice with 0.85% NaCl. Subsequently, the cells were resuspended and soaked with 0.85% NaCl containing 1% of formalin. Later, 24 h post-soaking at 4 °C, 100 µL of the mixture was collected and spread on BHI agar to confirm their non-viability. After obtaining the formalin-killed cells, all of them were centrifuged and washed with 0.85% NaCl, as above. This FKC or StrepKU-1 vaccine was kept in 0.85% NaCl containing 0.1% of formalin at 4 °C until further use.

### 2.3. Preparation of StrepKU-1-Loaded HNTs

Four types of bare HNTs, including H, HA, HC and HAC, were used to prepare the StrepKU-1-loaded HNTs. Briefly, the StrepKU-1 vaccine was combined with HNTs (H, HA, HC or HAC) at an optimized dosage of 15 mg mL^−1^. The mixtures were stirred for 3 h at room temperature. Finally, pellets of the StrepKU-1-loaded HNTs were collected by centrifugation at 6500× *g* for 20 min and 4 °C (Tomy, Tokyo, Japan) and stored at 4 °C for further mixing with feed pellets. All the StrepKU-1-loaded H, HA, HC and HAC were designated as HF, HAF, HCF and HACF, respectively.

### 2.4. Physical Characterization by Scanning Electron Microscopy (SEM)

Prior to the cell incubation, mica substrate (V1 grade, Ted Pella, Redding, CA, USA) was coated with 0.01% poly-L-lysine solution (PLL) (Sigma-Aldrich, St. Louis, MO, USA). After incubation for an hour at room temperature, the chemically coated mica was rinsed with deionized water and dried with nitrogen gas. The HF, HAF, HCF and HACF were diluted and incubated on the PLL-coated mica for an hour at room temperature. The unbound complexes were rinsed with deionized water, and the samples were dried with nitrogen gas. The samples were mounted on the sample holder with carbon tape and were coated with platinum for 50 s (Q150R sputter coaters, Quorum Technologies, East Sussex, UK). All the samples were imaged under a scanning electron microscope (FE-SEM SU8030, Hitachi High-Technologies, Tokyo, Japan) [35].

### 2.5. Acid, Base and Bile Salt Tolerance Profile of StrepKU-1-Loaded HNTs

To evaluate the releasing patterns of the HF, HAF, HCF and HACF under mimic digestive system conditions, the acid, base and bile salt tolerance analysis was conducted. Protein lysate from *S. agalactiae* serotype Ia and III was prepared by sonicating the bacterial cell suspension. The lysate was separated from the cell debris by centrifugation before being combined with HNTs at a 10:1 ratio. The performance of *S. agalactiae* proteins loaded in HNTs (Strep-loaded HNTs) was evaluated by 12% SDS -PAGE. The acid, base and bile salt tolerance and releasing characteristics of the Strep-loaded HNTs, H, HA, HC and HAC, were observed under the simulated pH and bile salt conditions [34].

### 2.6. In Vivo Efficacy Analysis of the StrepKU-1 Loaded HNTs

#### 2.6.1. Vaccination and Challenge Test

To prove the efficacy of orally administered HF, HAF, HCF and HACF against streptococcosis infection in tilapia, experimental fish were divided into 10 groups, including a mock group (fed with NaCl-mixed pellet), a positive control group (injected with StrepKU-1 vaccine), 4 groups of bared HNTs (fed with H-, HA-, HC- and HAC-mixed pellet) and another 4 groups of the HF, HAF, HCF and HACF (fed with the StrepKU-1 loaded H-, HA-, HC- and HAC-mixed pellet). Prior to starting vaccination, 60 fish (50  ±  5 g) of each treatment (20 fish per replicate) were transferred to glass tanks containing optimally aerated water at 30  ±  3 °C. All fish were fed with feed pellets twice daily.

After one week of acclimatization, 9 groups were fed with the freshly prepared feed pellets containing the vaccine (10^8^ CFU per gram) with approximately 3% of the weight of the fish at week 1 and week 3. The positive control group was intraperitoneally (IP) injected with the StrepKU-1 vaccine and was fed with commercial feed pellets daily. After a month post-vaccination, all remaining fish of each treatment (12 fish per tank in triplicate) were transferred to the new glass tanks and acclimatized for a week before being challenged with *S. agalactiae* serotype III (virulent strain) at 1 × 10^8^ CFU mL^−1^ [36]. Symptoms of infection and mortality were recorded for three weeks. The timetable representing the vaccination and challenge test is shown in Figure S1. Bacteria were isolated from the moribund fish using BHI agar and were subsequently confirmed by multiplex PCR based on the *cps* gene [36] to affirm the serotype of *S. agalactiae*. The efficacy of vaccines was demonstrated by cumulative survival and relative percentage survival (RPS). The normality and homoscedascity of the data distribution were verified by the Shapiro–Wilk test and skewness test. An analysis of paired two-sample *t*-tests for means was performed for statistical analysis, and *p*  <  0.05 was considered statistically significant. The SPSS program was used to conduct the statistical analysis.

#### 2.6.2. Antibody Titer Assay

Before taking blood, fish were anesthetized with eugenol. Blood samples were collected from the caudal vein of nine fish of each treatment at week 2 and week 4 (Figure S1). Fish sera were separated by centrifugation at 600× *g* for 15 min of 25 °C. An amount of 50 mL of 0.85% NaCl was added before adding 50 mL of the serum sample. Then, a two-fold serial dilution was performed until reaching 1/2048 times. Next, 50 mL of the FKC-Strep vaccine of each serotype diluted in 0.85% NaCl was added separately into the entire wells. The 1st column and the 12th column were marked as the positive and negative controls, respectively. The agglutination result was recorded after incubation at 37  °C for 24 h. Subsequently, the antibody titer graph was made to evaluate antibody production.

## 3. Results

### 3.1. Physical Observation of StrepKU-1-Loaded HNT Complexes by SEM Images

**Figure 1:**
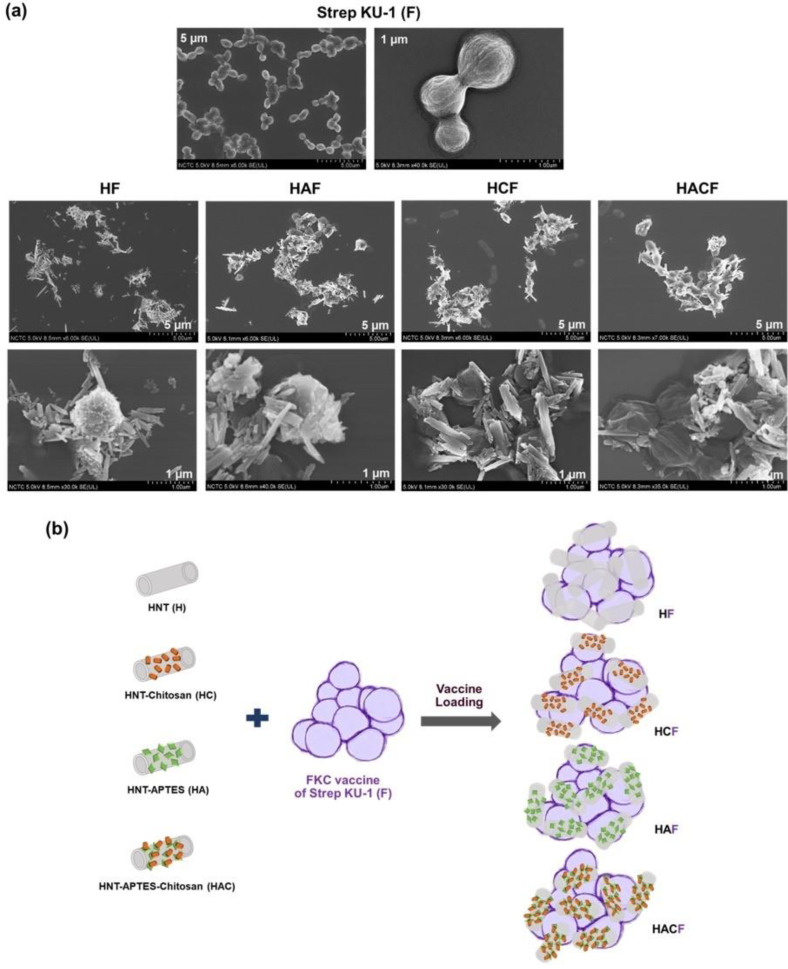
Scanning Electron Microscopy (SEM) observation and schematic diagram of Strep KU-1 and HNTs: (**a**) SEM images of the bared Strep KU-1 molecules (F) as well as the complexes of Strep KU-1 combined with HNTs (HF, HAF, HCF and HACF) at scale bars of 1 µm and 5 µm. (**b**) The schematic diagram proposing the surface binding characteristics of HNT moiety on the Strep KU-1 surface.

The morphology of the Strep KU-1-loaded HNTs was characterized by SEM analysis. They originated as Strep KU-1, the formalin-killed cells, which were the spherical cocci form of the *S. agalactiae* cells in which the bacterial cells remained intact even after they were inactivated by formalin. Interestingly, after adding HNTs, all of them were adhered and covered the bacterial cell surface (Figure 1a). However, the degree of HNT conjugation could not be elaborated by SEM analysis. The entrapment profile between Strep KU-1 and HNT moiety was proposed (Figure 1b).

### 3.2. Acid, Base and Bile Salt Tolerance

To evaluate the ability of the HNT-loaded vaccines in releasing the formalin-killed cell vaccines in the mimic digestive tract, the HNT-loading *S. agalactiae* protein lysate was prepared from *S. agalactiae* serotype Ia and III (Figure S2). After loading the bacterial protein lysate to the HNTs, the same protein patterns were observed in both protein-loaded HNTs and bacterial protein lysates. Importantly, a few protein lysates remained in the supernatant (unbound fraction), demonstrating the success in loading target proteins in HNTs, and almost all the protein loading could be bound to the HNTs at the performed ratio. However, the loading pattern and capacity among H, HA, HC and HAC appeared similarly (Figure S2b). This indicated that unmodified HNTs and modified HNTs loaded with *S. agalactiae* protein lysate could be readily used for determining acid, base and bile salt tolerance tests.

**Figure 2:**
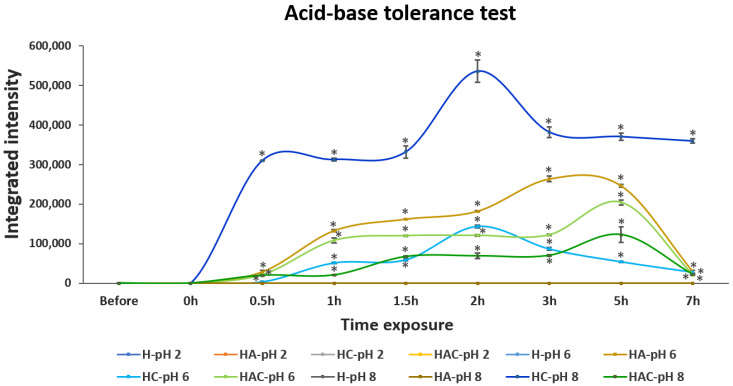
The integrated intensity profile of *S. agalactiae* protein-lysate-loaded HNTs while exposing them to acid–base solutions at pH 2, pH 6 and pH 8 for 7 h. H, HA, HC and HAC indicate the protein releasing profiles of HNT, HNT-APTES, HNT-Chitosan and HNT-APTES-Chitosan, respectively. Notably, the releasing profile of Strep proteins in pH 2 of all Strep-loaded HNT formulations, including H-pH 6, H-pH 8 and HA-pH8, were non-detectable (n.d.). Statistical analysis was performed via a paired two-sample *t*-test for an analysis of means with *p* < 0.05. “*” indicates that the HA-pH6, HC-pH6 and HAC-pH6 were significantly different from H-pH6 and that HC-pH8 and HAC-pH8 were significantly different from H-pH8.

The releasing and tolerance profiles in acid–base conditions at pH 2, pH 6 and pH 8 were examined. *S. agalactiae* protein lysate was not detectable in both solutions and pellet fractions after being treated in pH 2 (Figure 2 and Figures S3a). This may have resulted from the degradation of proteins in rather harsh conditions. *S. agalactiae* protein lysate could be observed in pH 6 and pH 8 conditions. In the case of protein-lysate-loaded HA and HAC, the gradual releasing of protein lysate was detected, and the highest number of proteins was observed after 5 h of exposure at pH 6. The releasing of the protein decreased at pH 8. However, protein-lysate-loaded HC was detected at pH 6–8, especially at 2 h of exposure. Moreover, the H type did not exhibit the releasing profile in any simulated pH environment (Figure 2 and Figure S3b,c). However, most *S. agalactiae* protein-lysate-loaded HNTs remained intact in the HNTs at both pH 6 and pH 8 conditions (Figure S3b,c).

**Figure 3:**
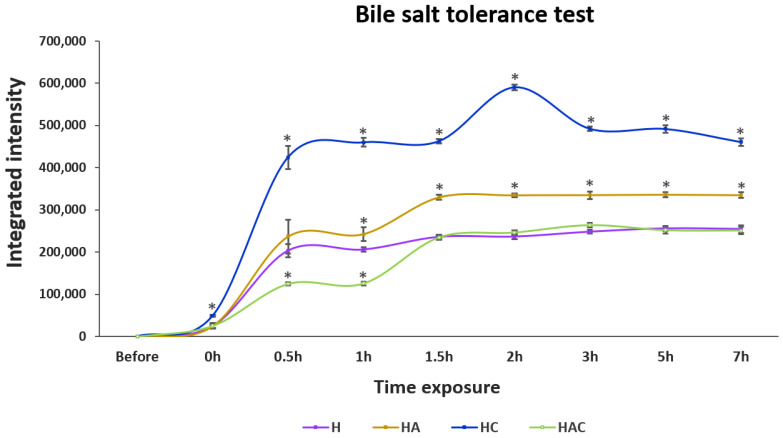
The integrated intensity profile of *S. agalactiae* protein-lysate-loaded HNTs exposed to 0.3% bile salt solution for 7 h. H, HA, HC and HAC represent the protein-releasing profiles of HNT, HNT-APTES, HNT-Chitosan and HNT-APTES-Chitosan, respectively. Statistical analysis was performed via a paired two-sample *t*-test: for an analysis of means with *p* < 0.05. “*” indicates a significant difference between unmodified (H) and other modified HNTs (HA, HC and HAC) at each time point.

In bile salt conditions, HC showed the highest amount of released proteins, followed by HA, H and HAC after 7 h of exposure (Figure 3 and Figure S4). Remarkably, the releasing profile of the H type could not be noticed when exposed to pH 2–8 solution, but it showed releasing in the bile salt solution, as with other HNTs.

### 3.3. Specific Immunity of Fish Fed with the HNT Nano-Delivery System

**Figure 4:**
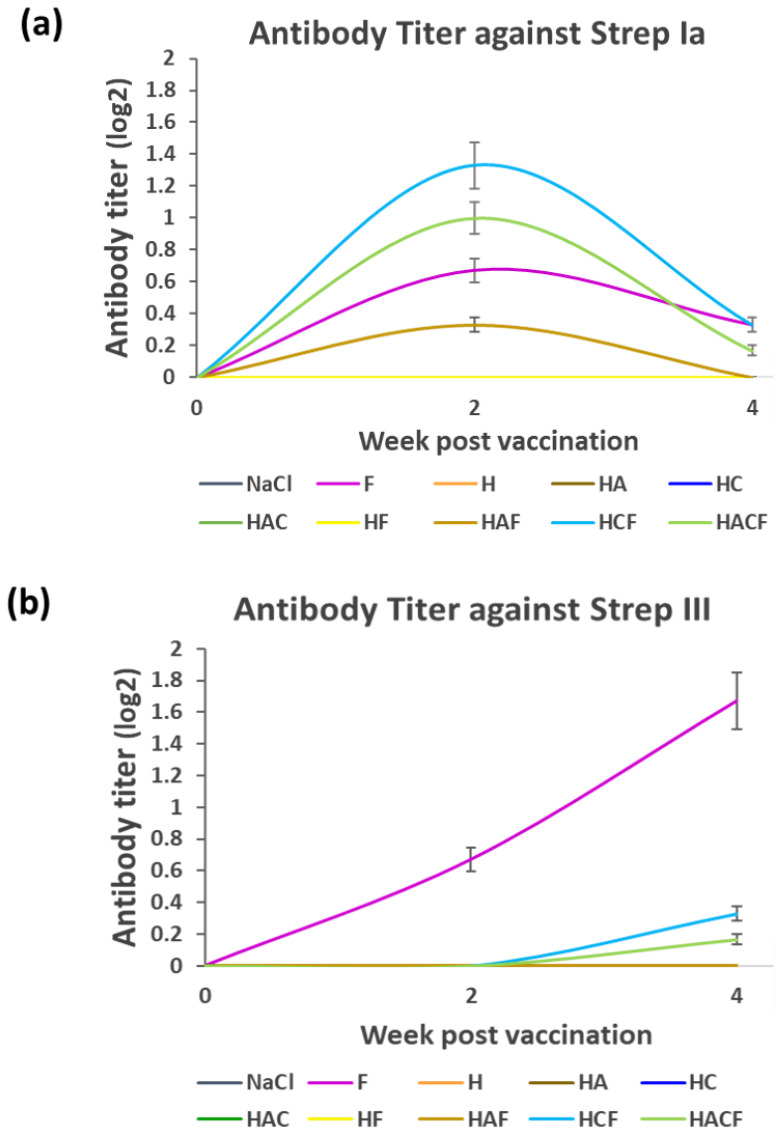
Antibody titer determination at week 2 and 4 post-vaccination: (**a**,**b**) the antibody titer levels against *S. agalactiae* serotype Ia and III (Strep Ia and III), respectively. Data are represented as the means ± SDs (n = 3; n represents three replicated aquarium tanks). F: fish injected with the StrepKU-1 vaccine marked as a positive control, NaCl: fish fed with 0.85% NaCl solution marked as a negative control.

The antibody titer was monitored from the serum of StrepKU-1-loaded-HNT-fed fish at week 1 and week 3. Due to the StrepKU-1 vaccine containing both *S. agalactiae* serotypes Ia and III, the antibody titer was separately determined against both serotypes. In the case of *S. agalactiae* serotype Ia (Strep Ia), the antibody titer level of the fish fed with the HAF, HCF and HACF was increased at week 2 but decreased at week 4, which was similar to the StrepKU-1-injected group (marked as F), as shown in Figure 4a. Noticeably, the HCF feeding group showed the highest level of specific antibody production than the others, including the FKC (F)-injected group. On the other hand, the titer was undetectable in other remaining groups, including the negative control (NaCl group).

The antibody titer profile against *S. agalactiae* serotype III (Strep III) was remarkably different from the Strep Ia profile (Figure 4a). The pattern of the antibody titer of the F-injected group was increased at week 2 and exponentially extended until week 4 of sampling. The antibody titers in HCF and HACF were later gradually increased at week 4. However, none of the specific antibodies could be detected in other groups (Figure 4b). The data spreadsheet of average mean value of antibody titer level against *S. agalactiae* serotype Ia and III (Strep Ia and III) was shown in Figure S5.

### 3.4. Efficacy Analysis of the StrepKU-1-Loaded HNTs against Streptococcosis

Eventually, to examine the feasibility of unmodified and modified HNTs for delivering the vaccine, their efficacies were evaluated based on an in vivo experiment. After oral administration for a month and affirming the production of specific antibodies against *S. agalactiae*, the experimental fish were challenged with *S. agalactiae* (serotype III).

**Figure 5:**
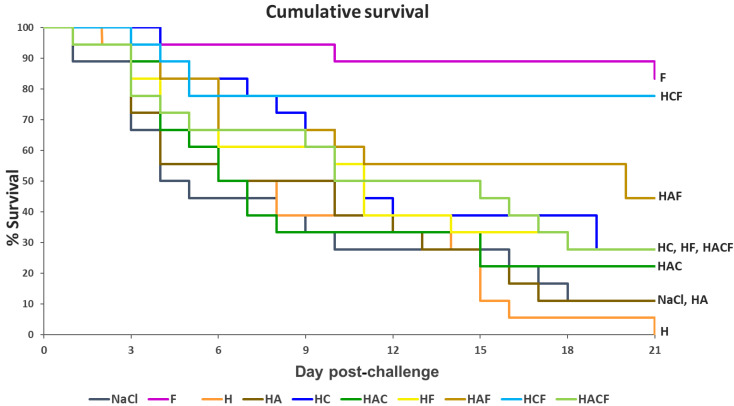
The Kaplan–Meier graph representing the cumulative survival of tilapia (*Oreochromis* sp.) after being challenged with *S. agalactiae* serotype III at 1 × 10^8^ CFU.mL^−1^ for three weeks.

Through injection, the StrepKU-1 vaccine (marked as F) demonstrated the highest vaccine efficacy, with an 83.33% survival rate. Through oral administration, among the tested groups, HCF provided the highest protective efficacy with a survival rate of 77.78%, followed by the HAF group with a rate of 44.44%. Moreover, a similar fish survival rate was observed in the HF and HACF groups with a rate of 27.78% (Figure 5). Interestingly, the survival rate of fish administered with HCF was dramatically reduced within 5 days post-challenge and was still retained thereafter. This evidence was not observed in other challenging cases, including the StrepKU-1 injection group, where the survival rate gradually reduced (Figure 5).

**Figure 6:**
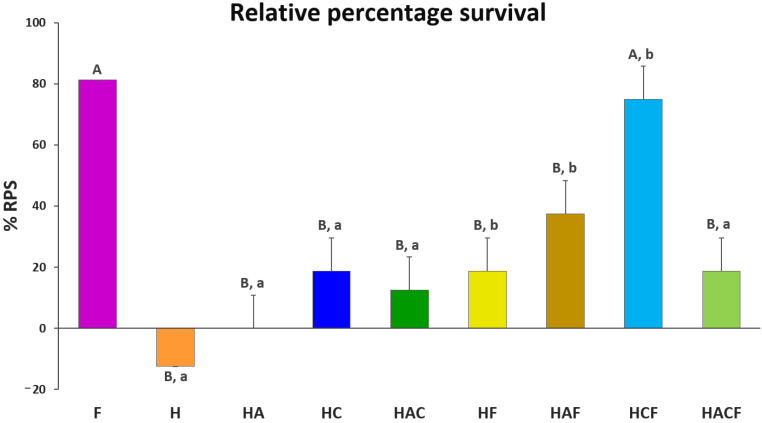
Relative percentage survival (%RPS) of the experimental groups based on that of the control NaCl group at 21 days post-challenge. Data are represented as the means ± SDs (n = 3; n represents three replicated aquarium tanks). Statistical analysis was performed via a paired two-sample *t*-test for an analysis of means with *p* < 0.05. The capital letters above the bars indicate the significant differences between the F group (StrepKU-1 injection) with others. Moreover, the small letters indicate significant differences between those with and without loading the StrepKU-1 for each pair of materials (H vs. HF, HA vs. HAF, HC vs. HCF and HAC vs. HACF).

The %RPS of all vaccinated groups were compared with the injected StrepKU-1 vaccine (81.25 ± 0.00%). Importantly, the %RPS of HCF (75.00 ± 10.83%) showed no significant difference with the injected StrepKU-1 vaccine. This observation was different from other StrepKU-1-loaded HNTs, in which the %RPS of the HF and HACF groups showed the same level of %RPS (18.75 ± 10.83%), whereas the HAF group was 37.50 ± 10.83%, and all of them showed significantly lower %RPS compared with the injected StrepKU-1 vaccine and the HCF-administered group (Figure 6).

The ability for disease prevention by a bare HNTS delivery system was demonstrated. It was shown that all StrepKU-1-loaded-HNT-treated groups exhibited higher %RPS than bare HNTs after *S. agalactiae* infection. Notably, the HC–HCF groups demonstrated the highest significant difference of %RPS by 56.25%, which was similar to the H–HF and HA–HAF groups. However, only the HAC–HACF groups revealed no significant difference in %RPS (the lowest difference was at 6.25%) (Figure 6). This result demonstrated that the HNT nano-delivery system was not able to prevent streptococcal disease infection, and the prevention of this disease arose from the loading of the vaccines.

During the challenge test, the moribund fish with clinical signs of streptococcosis (Figure S6a) were noticed, especially in the negative control group and in the fish fed with the bare HNTs. *S. agalactiae* serotype III could re-isolate from challenged fish, as confirmed by the multiplex PCR (Figure S6b).

## 4. Discussion

Although most commercialized vaccines being acceptable globally are injection vaccines, they still cause adverse effects and several limitations, particularly for manually injecting in large-scale farmed fish. Thus, an oral vaccine, or an alternative vaccine type, should be formulated against critically pathogenic diseases, including streptococcosis disease impacting tilapia throughout the world. To date, there is still a small amount of innocuous and effective oral vaccines that have been approved for utilization both in humans and animals [10,37]. However, recently, the development of oral feed-based vaccines has been focused upon for them to become an ideal approach for preventing and controlling fish infectious diseases. Up to now, oral vaccines based on live attenuated *S. agalactiae*, *E. coli*, *Bacillus subtilis* and *Lactococcus lactis* expressing the *S. agalactiae* immunogenic proteins have been studied against streptococcosis disease in tilapia hosts [31,38,39,40]. Nevertheless, their relative percentage survival results still fluctuated, and some formulations also depended on oil adjuvants [27,31]. Consequently, the development of fish oral vaccines based on encapsulation and entrapment strategies should receive more concern.

In the past decades, nanotechnology has become a promising tool for delivering various kinds of biologics and active compounds, such as antimicrobial agents, enzymes and other drugs which can be applied for different purposes [41,42,43]. Until now, several smart nanocarriers were fabricated and applied from both natural and synthetic materials. Halloysite nanotubes (HNTs) are one of the referred targets as efficacious delivering substances for an oral delivery system. As a result of their low toxicity, good biocompatibility, low price and unique structure and surface [44], therefore, HNTs can provide a number of desirable or specific characteristics for exploitation. The modification of the outer and inner surfaces of HNTs has been reported to enhance their biocompatibility properties [44,45,46], which can interact with the surfaces of endothelial cells layered on animal digestive tracts through ionic interactions [47]. Alternatively, their hollow features also enable HNTs to encapsulate more loading molecules [16,45].

The tuning of HNT surfaces with organosilane and biopolymer resulted in different amounts of BSA adsorption. Conversely, the current study proposed that the capacity of both unmodified and modified HNTs for carrying cell lysate proteins of *S. agalactiae* bacterium seemed to be similar. However, their releasing profiles among variable pH and bile salt solutions were uniquely distinct. Consistency with the others suggested that the loading ability of biological molecules not only depended on the HNT surfaces but was also affected by characteristics of the loaded substances [48]. Most commonly, the positively and negatively charged molecules performed electrostatic interactions with HNTs’ outer and inner surfaces, respectively [49]. Additionally, the grafting of HNTs with polymers or others allowed more types of bonding, such as the covalent bonds, hydrogen bonds and Van der Waals forces, exposing modified HNT surfaces and their loadings [50,51,52].

Unfortunately, the proteins of our bivalent vaccine (formalin-killed cells–*S. agalactiae*) were invisible in SDS-PAGE analysis, which was similar to the FKC-*E. coli*, but some antigenic proteins could be detected by Western blot analysis [53]. It may explain that, during inactivation by formalin in the vaccine preparation process, the bacterial proteins were denatured and fixed in their cell membranes; consequently, protein contents were retained in the bacterial cell structures [54]. Therefore, whole-cell bacteria were not able to migrate through the porous matrix of the SDS-PAGE and became stuck in the wells [53]. Hence, to evaluate the binding capability of HNTs to FKC, *S. agalactiae* protein lysate was employed instead of whole-cell FKC. However, the achieved entrapment between the streptococcal bivalent vaccine and HNTs was verified by SEM observation. Correspondingly, the obtained results seemed to resemble the SEM images, showing that halloysite nanotubes were deposited on the cell surfaces of yeast, *Saccharomyces cerevisiae* [55].

In this study, we aimed to generate an alternative oral delivery vaccine for fish. Herein, an in vitro acid, base and bile salt analysis was used to evaluate the compatibility of the condition of HNT delivering the whole-cell inactivated vaccines through the digestive tract. Among the HNTs and their derivatives, HC demonstrated an excellent releasing profile under the simulated physiological conditions. The pH of the stomachs and intestines of tilapia ranged from 2.23–6.66 and 6.27–7.46, respectively [56]. Therefore, it can be mentioned that HCF may resist lower pH in the stomach and prevent biologics from degradation by gastric digestion. This action may arise from the physical structure of HC, in which surface chitosan can control and prevent biologics in a strong acidic condition before later releasing in the intestine [16,57,58].

To affirm the capability of H and their derivatives (HA, HC and HAC) in carrying the bivalent streptococcosis vaccine and delivering the vaccine through oral administration, specific immunity against virulent *S. agalactiae*, serotype I and serotype III, was evaluated from the sera of orally vaccinated fish. HCF and HACF have shown greater specific immunity to serotype Ia than the control (bivalent vaccine injection group), whereas only the control group showed increasing immunity to serotype III. This observation resembled the greater response of the bivalent vaccine to serotype Ia than to serotype III (data not shown), which was affected by the higher number of antigenic proteins in serotype Ia [32]. Moreover, the route of the oral vaccine in inducing immunity could explain the hydrolysis and releasing of antigens in the digestive tract, followed by host immune system recognition. Once the HCF passes from the stomach to the intestine, hydrolysis promptly takes place, leading to a significant shift from the protein to short soluble polypeptides or oligo- and dipeptides [56]. Afterwards, those peptides are absorbed as a result of activating specific immunity through gut-associated lymphoid tissue (GALT) immunity [59]. This observation confirmed the achievement in inducing immunity in orally vaccinated fish, and HC and HCF were suggested to be potent delivery agents for oral vaccination in fish.

After determining the delivery potency of the vaccine-based HNT materials, the highest survival rate of infected fish was observed in the HCF-treated group, which was not significantly different from the bivalent vaccine injection group. This suggests that these modified nanotubes exhibited suitable properties in delivering inactivated vaccines to the immune recognition site. Regarding the obtained result, it could also be implied that our formulated system can protect the vaccine from gastric digestion, and the vaccine could release in the intestine, where the GALTs are presented. This investigation is supported by the evidence that chitosan-modified HNTs can play as efficient drug carriers to treat several health problems [48,58,60,61]. Moreover, it can be noted that, even though HCF had higher protection against virulent *S. agalactiae* (serotype III) than H, HA and HAC, the specific antibody against serotype III was much lower than serotype Ia.

However, the bivalent vaccine loading capacity among H derivatives was not different, as seen from the SEM analysis. HCF was found to induce better specific immunity and to provide the highest disease protection than others. It can be noted that the modification of HNTs results in enhancing immunostimulation and later protection to a specific disease. In this study, chitosan was remarkably noticed as a proper biomolecule for derivatizing nanotubes. The biological function of chitosan and its derivatives (such as glycated chitosan, sulfated chitosan and trimethyl chitosan) have contributed to the immunostimulatory effect, and thus, it has been applied as a vaccine adjuvant and biologics delivery system [62,63,64,65]. However, even though HACF also contains chitosan, its immunostimulation and protection were not as similar as HCF. Most likely, HACF has different physical properties such as surface charge and the amount of chitosan from HCF. Depending on the determination of their zeta potential value [34], the HAC may contain lower amounts of chitosan than the HC that affects their adsorption efficacy to biologic molecules [66]. As a result, the immune stimulation of the HACF-vaccinated fish was diminished, and disease protection was consequently reduced when compared with HCF-treated fish.

HNTs in animal use should be considered for safety. Although HNTs provide numerous advantages including being eco-friendly, having a low cost and having biocompatibility, their toxicity threshold has been under discussion [67,68]. Based on the previous studies, most of them stated that using HNTs as a delivery system is quite safe for animals and humans [69].

However, some publications have proposed that HNTs can induce side effects depending on their types and their targeted host organisms. Toxic investigations in murine models revealed that reversible inflammation within the small intestine was detected after the oral uptake of high-dose HNTs at 50 mg kg^−1^ of body weight for 30 days daily. However, no harmful effects were found when feeding with a lower dose (5 mg kg^−1^ body weight), and their weight still normally increased [70]. In another case, when the embryos of the zebrafish were treated with HNTs at a high concentration (25 mg kg^−1^), not only did they have no considered impacts on the treated embryos, but they also stimulated their hatchability. Moreover, the ingested HNTs were gradually excreted from the zebrafish larvae’s digestive tract by their gastrointestinal metabolism [71].

Taken together, it can be concluded that the required effect of the delivery system is the ability to release the vaccine at target sites and the ability of the host to take up the antigenic molecules to induce the specific immunity. Superior adhesion to endothelial cells was achieved by ionic interactions between the cationic surface on chitosan-modifying HNTs and on the anionic surfaces of endothelial cells. This interaction can promote and enhance cell/ biologics docking to the cells layered on the surface of the intestine and can transfer mucosal immune activity. Our study supports the utilization of HNTs as a biologics delivery system, including oral vaccines for fish. Remarkably, our oral HCF formulation provided no significantly different efficacy with the injection administration by the same FKC vaccine.

## 5. Conclusions

To overcome the limitations of tedious work on vaccine injections for aquatic animals, the novel nano ‘biologics’ delivery vehicle was developed from nano clay HNTs. The functionalized surfaces of HNTs improved the efficiency of vaccine loading and immune stimulation, which later affected the disease protection, as proven by an in vivo analysis. Interestingly, it can be mentioned that chitosan-functionalized HNTs (HCF) provided promising whole-cell inactivated vaccine-loading properties, protected them from the harsh digestive environment and promoted their bioavailability to GALTs in the host’s digestive tract. Importantly, under controlled conditions, the efficacy of our orally bivalent vaccine remained high, similar to the injection method. Taken together, the findings highlight that our oral vaccine delivery system can feasibly facilitate the fish vaccination system for controlling and preventing bacterial disease outbreaks in fish cultivation. This oral administration system can reduce the obstacles to fish vaccination and can support the sustainability of aquaculture.

## Data Availability

All data relevant to the study are included in the article.

## Supplementary Materials

The following supporting information can be downloaded at https://www.mdpi.com/article/10.3390/vaccines10081180/s1, Figure S1: The timetable represents experimental designs of vaccination and challenge test, Figure S2: Protein pattern of *S. agalactiae* protein lysate and *S. agalactiae* protein lysate-loaded HNTs, Figure S3: The 12% SDS-PAGE analysis showing the acid-base tolerance profile of the *S. agalactiae* protein lysate-loaded HNTs after exposure to artificial acid-base solutions, Figure S4: 12% SDS-PAGE analysis of the bile salt tolerance profile of the *S. agalactiae* protein lysate-loaded HNTs after 7 h, Figure S5: The data spreadsheet of average mean value of antibody titer level against *S. agalactiae* serotype Ia and III (Strep Ia and III), Figure S6: The agarose gel electrophoresis demonstrating the multiplex PCR to confirm *S. agalactiae* serotype III infection and the noticeable symptoms after infection.
