# Supplementary material for: A Novel Efficient Piscine Oral Nano-Vaccine Delivery System: Modified Halloysite Nanotubes (HNTs) Preventing Streptococcosis Disease in Tilapia (Oreochromis sp.)
Source: Vaccines (Basel). 2022 Jul 25;10(8):1180. doi: 10.3390/vaccines10081180 (PMC9331641; doi:10.3390/vaccines10081180)
Supplement: Supplementary file 1 [file vaccines-10-01180-s001.zip › vaccines-1759196-supplementary.pdf]

Supplementary Figures

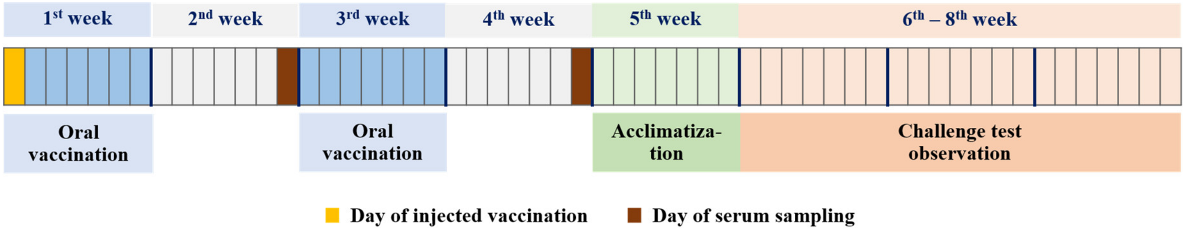

Figure S1. The timetable represents experimental designs of vaccination and challenge test. The oral vaccinated groups were orally fed with HNTs with and without Strep-KU1 vaccine at the 1st and 3rd week (highlighted with blue color), while the injected group was vaccinated on day 1 (designated with orange color). Meanwhile, a brown color indicates the day of serum sampling in week 2 and week 4. The experimental fish were acclimatized for a week (at week 5) as highlighted in green color. Also, an orange color represents the duration of the challenge test (at 6th to 8th week).

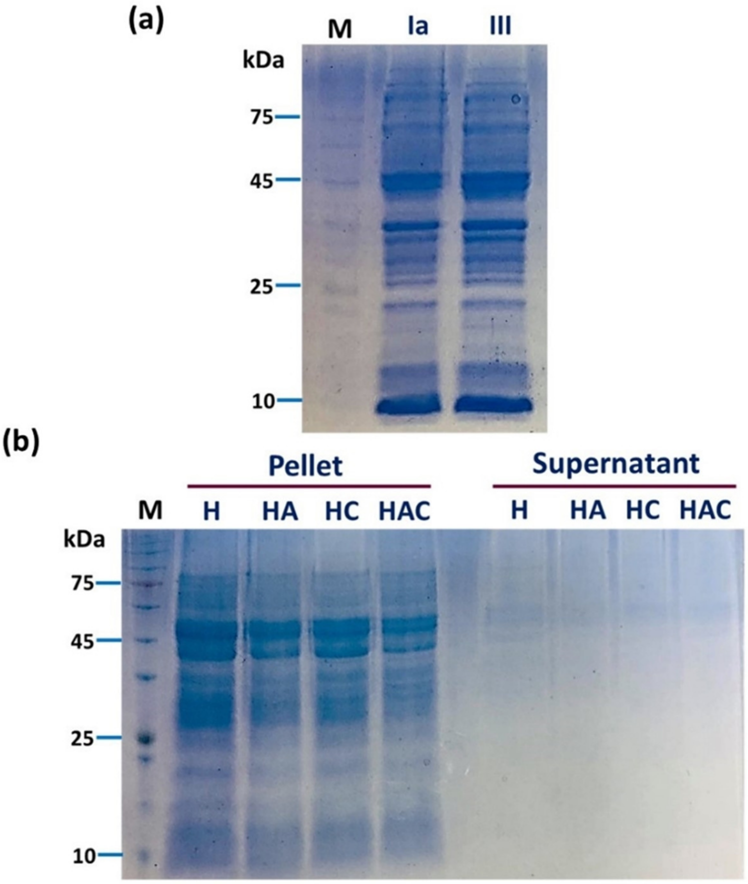

Figure S2. Protein pattern of *S. agalactiae* protein lysate and *S. agalactiae* protein lysate–loaded HNTs. (**a**) whole-cell protein profile of *S. agalactiae* serotypes Ia and III, (**b**) profile of the *S. agalactiae* protein lysate-loaded HNTs. Pellet represented the proteins that bound to the HNTs, supernatant exhibited the remaining proteins in solution. H, HA, HC, and HAC stranded for HNT, HNT-APTES, HNT-Chitosan, and HNT-APTES-Chitosan, respectively. (M = protein marker).

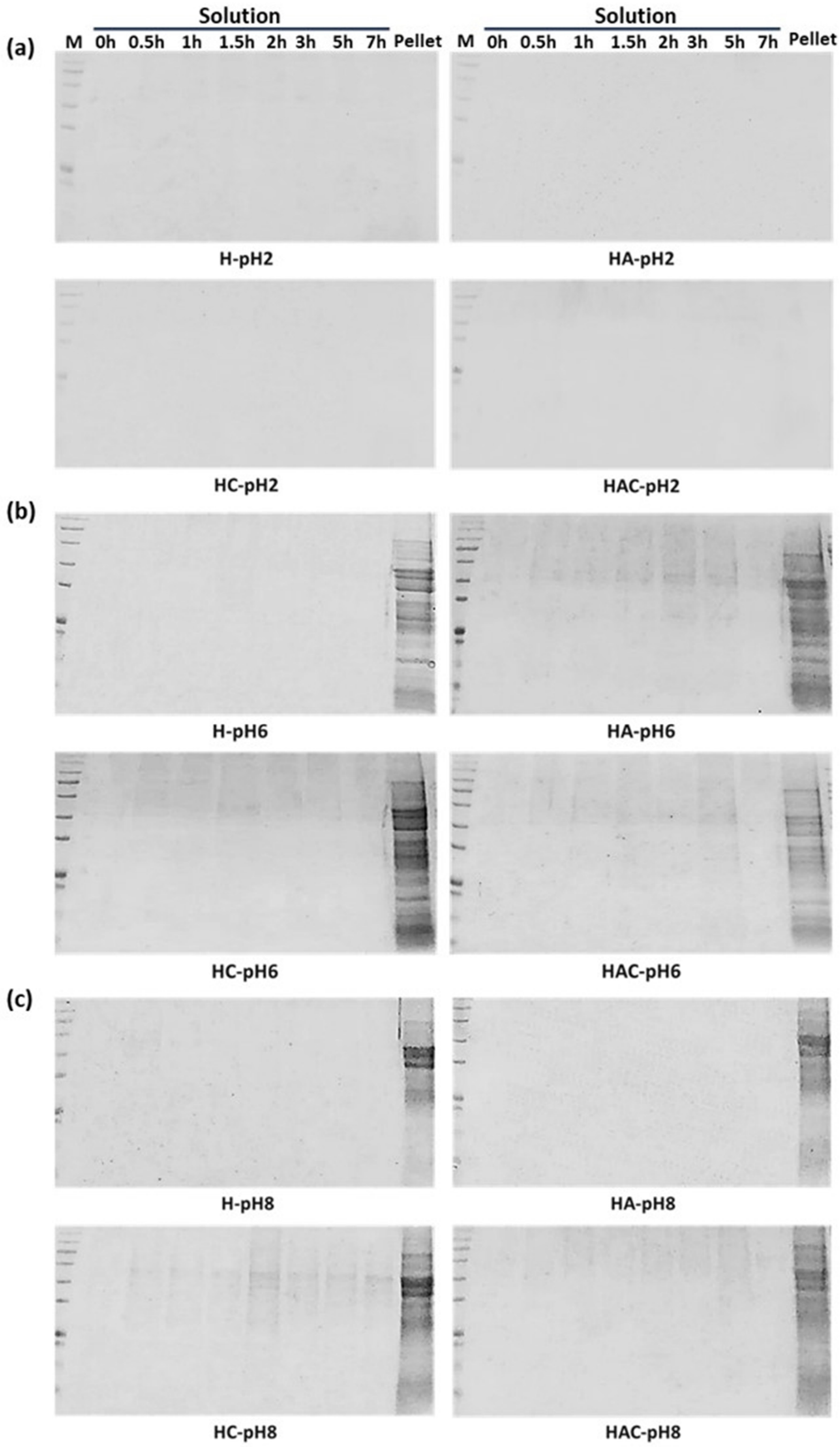

Figure S3. The 12% SDS-PAGE analysis showing the acid-base tolerance profile of the *S. agalactiae* protein lysate-loaded HNTs after exposure to artificial acid-base solutions. (**a**), (**b**) and (**c**) represented the releasing patterns at pH 2, pH 6, and pH8, respectively. The detectable band in the solution was the releasing *S. agalactiae* protein lysate, while in the pellet was the remaining proteins on HNTs particles.

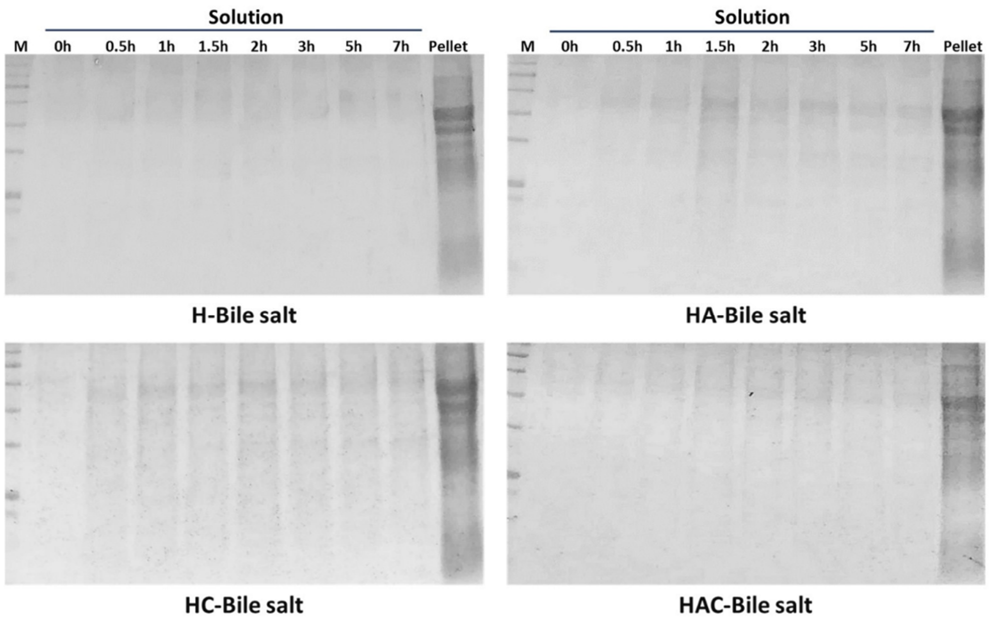

Figure S4. 12% SDS-PAGE analysis of the bile salt tolerance profile of the *S. agalactiae* protein lysate-loaded HNTs after 7h. The observed protein bands in the solution were the releasing Strep proteins, whereas in the pellet was the remaining proteins on HNTs.

Week	NaCl	F	H	HA	HC	HAC	HF	HAF	HCF	HACF	Treatment
**0**	0	0	0	0	0	0	0	0	0	0	
**2**	0	0.67	0	0	0	0	0	0.33	1.33	1	
**4**	0	0.33	0	0	0	0	0	0	0.33	0.167	
	**Average mean of antibody titer value against Strep Ia**										

Week	NaCl	F	H	HA	HC	HAC	HF	HAF	HCF	HACF	Treatment
**0**	0	0	0	0	0	0	0	0	0	0	
**2**	0	0.67	0	0	0	0	0	0	0	0	
**4**	0	1.67	0	0	0	0	0	0	0.33	0.167	
	**Average mean of antibody titer value against Strep III**										

Figure S5. The data spreadsheet of average mean value of antibody titer level against *S. agalactiae* serotype Ia and III (Strep Ia and III). F: fish injected with the StrepKU-1 vaccine marked as a positive control, NaCl: fish fed with 0.85% NaCl solution marked as a negative control. H, HA, HC, and HAC groups: fish fed with H, HA, HC, and HAC-mixed pellet, respectively. HF, HAF, HCF, HACF groups: fish fed with the StrepKU-1 loaded H-, HA-, HC- and HAC-mixed pellet.

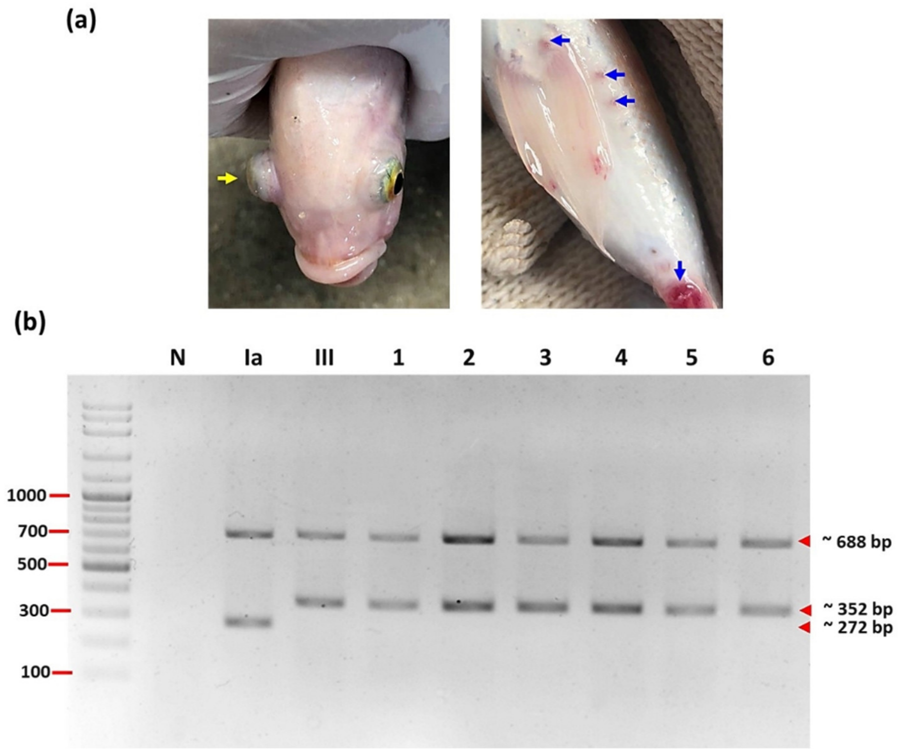

Figure S6. The agarose gel electrophoresis demonstrating the multiplex PCR to confirm *S. agalactiae* serotype III infection and the noticeable symptoms after infection. (**a**) The exophthalmia and abscess symptoms were indicated at yellow and blue arrows, respectively. (N = negative, Ia = positive for *S. agalactiae* serotype Ia, III = positive for *S. agalactiae* serotype III, 1-6 = number of fish samples). (**b**) The specific bands of *S. agalactiae* serotype III were indicated at approximately 688 bp and 352 bp.
